# The nematicide emamectin benzoate increases ROS accumulation in *Pinus massoniana* and poison *Monochamus alternatus*

**DOI:** 10.1371/journal.pone.0295945

**Published:** 2023-12-21

**Authors:** Fengzhu Liu, Shunde Su, Jie Chen, Qinghua Xu, Gaofei Song, Yuguang Dong, Xiangqing Jiang, Daoshun Chen, Yu Fang, Jian Li, Chentao Lin, Jun Su, Shouping Cai

**Affiliations:** 1 Basic Forestry and Proteomics Research Center, College of Forestry, Fujian Provincial Key Laboratory of Haixia Applied Plant Systems Biology, Fujian Agriculture and Forestry University, Fuzhou, China; 2 Fujian Academy of Forestry, Key Laboratory of National Forestry and Grassland Administration on Timber Forest Breeding and Cultivation for Mountainous Areas in Southern China, Fuzhou, China; 3 Syngenta (China) Investment Co. Ltd., Shanghai, China; 4 Shaxian Guanzhuang State-Owned Forest Farm, Sanming, China; 5 Institute of Soil Fertilizer, Fujian Academy of Agricultural Sciences, Fuzhou, China; University of Agriculture Faisalabad, PAKISTAN

## Abstract

Pine wilt disease (PWD) is caused by the pine wood nematode (PWN, *Bursaphelenchus xylophilus*) and transmitted by a vector insect, the *Monochamus alternatus*. The PWN has caused much extensive damage to pine-dominated forest ecosystems. Trunk injection of emamectin benzoate (EB) has been found to be the most useful protective measure against the PWN, due to its low effective dose and long residence time in the field. However, the interactions between EB and the host or the environment remain largely unknown, which limits the efficacy and stability of EB in practical field settings. In this study, we investigated the impact on PWN from EB injection for both adult and young host plants (*Pinus massoniana*) by taking a multi-omics (phenomics, transcriptomics, microbiome, and metabolomics) approach. We found that EB injection can significantly reduce the amount of PWN in both living adult and young pine trees. Additionally, EB was able to activate the genetic response of *P*. *massoniana* against PWN, promotes *P*. *massoniana* growth and development and resistance to Pine wilt disease, which requires the presence of PWN. Further, the presence of EB greatly increased the accumulation of reactive oxygen species (ROS) in the host plant in a PWN-dependent manner, possibly by affecting ROS-related microbes and metabolites. Moreover, we uncovered the function of EB limiting the consumption of *P*. *massoniana* by the JPS. Based on biochemical and gut microbial data, we found that EB can significantly reduces cellulase activity in JPS, whose transcription factors, sugar metabolism, and the phosphotransferase system are also affected. These results document the impact of EB on the entire PWD transmission chain through multi-omics regarding the dominant pine (*P*. *massoniana*) in China and provide a novel perspective for controlling PWD outbreaks in the field.

## 1. Introduction

Pine wilt disease (PWD) is caused by the pine wood nematode (PWN) *Bursaphelenchus xylophilus* which relies on vector insects to infect hosts [1–4]. The PWN is responsible for great economic and ecological damage to *Pinus* spp. forests and plantations worldwide, especially in southern China, where the largest PWN-damaged area exists [5, 6]. Effective prevention measures are crucial to PWD control [7]. In the last decade, tremendous efforts have been paid to developing effective strategies for mitigating PWD, including high-throughput PWD prediction and forecasting approaches [4]; the biological control of the PWN and its key vector, the Japanese pine sawyer (JPS, *Monochamus alternatus*) [8–12]; removing and sterilizing PWD-killed trees in forest stands [13–15]; and breeding PWN-resistant pine trees as well as nematicide implantation in the trunks of pine trees [16]. Yet the accelerated spread of PWD in recent years has spurred the need to develop more effective preventive measures against it [17].

The primary target of current PWD control measures is the PWN, whose most effective direct control is via trunk injection of powerful nematicides [17]. These include natural products, such as essential oils and volatile compounds from the host plants [17, 18], or (hemi)synthetic chemical compounds (e.g., emamectin benzoate, mesulfenfos, abamectin, iodoindoles, and acetamiprid) [14, 19]. Among them, emamectin benzoate (EB) has emerged as the most useful compound for trunk injection against the PWN, because it has a low effective dose, is environmentally friendly, and has a longer residence time (i.e., at least 3 years) in the field [14, 20]. Additionally, interactions with the host plant and the ecological environment of nematicides against the PWN have been explored and applied [14, 16, 19, 21], which has highly improved their field efficiency. Unfortunately, the interactions between EB and different hosts—especially for the dominant pine tree species, *Pinus massoniana*, in Southern China—and the environment are still largely unknown, limiting its efficacy and stability in the field.

Population outbreaks by the PWN disturbs the physiological and biochemical responses of the host pine tree [8, 22], as well as the symbiotic microbiota of the host plants [8, 23]. Nematicides are capable of reducing these PWN-caused impacts to the host plant by eliminating the amount of PWN in the latter [19, 24]. Moreover, previous studies have shown that EB can also damage the insect if consumed by it, including JPS [19, 24]. Given these considerations, we hypothesized that not only does EB injection decrease the PWN amount to augment the resistance response of *P*. *massoniana*, but it also could adversely affect the vector insect (JPS) via its diet.

In this study, we investigated the impact of EB injection on both the PWN and the host pine tree by using a multi-omics approach (phenomics, transcriptomics, microbiome, and metabolomics). We also explored the dietary impact of EB on the vector insect JPS. Accordingly, we documented for the first time the impact of EB on the entire transmission chain of PWD. This study provides a novel perspective for further improving the efficiency of EB, and a new approach to field control of PWD.

## 2. Material and methods

### 2.1 Plant material and growth conditions

For the laboratory experiments, 2-year-old seedlings of *P*. *massoniana* were harvested from a nursery garden in a Shaxian Guanzhuang state-owned forest farm in Sanming, Fujian, China (26.5603°N, 117.7455°E). They were placed in growth chambers (light: dark = 16 h: 8 h, 70% humidity, 28°C in light and 24°C in dark) for 2 months before the experimental treatments commenced.

The field experiments were carried out on the same state-owned farm. For this, the study area consisted of a 76-hectare, 15-year-old pure *P*. *massoniana* plantation, whose annual average temperature and precipitation are 19.9°C and 1375.2 mm, respectively. Approximately 16% of the pine trees have died from PWN infection within the last 5 years. The entire area was divided into seven plots according to geographical conditions (i.e., slope position, slope direction, slope gradient, and leaf area index) and infection conditions (S1 Fig), with the plots designated as 1–7; plots 1–5 and 7 were treatment plots with a total of n = 898 individuals, while plot 6 was the control (n = 118 individuals). In May 2022, 898 trees in the treatment group were injected with EB. According to their overall infestation condition, these trees were divided into six categories; *treatment plots*: 284 plants with more PWN (> 10 000 nematodes), 325 plants with less PWN (< 4000 nematodes), and 289 plants without PWN; *control plot*: 35 plants with more PWN (>10 000 nematodes), 42 plants with less PWN (< 4000 nematodes), and 41 plants without PWN.

### 2.2 Inoculation of pine wood nematode

PWN adults were cultured at a constant temperature of 25°C using *Pestalotiopsis* as previously described [8]. Next, PWN was isolated from media containing PWN using the Bellman funnel method, sterilized by centrifugation, and concentrated. 1 mL of a PWN inoculation solution (5000 individuals/mL) was inoculated into *P*. *massoniana* seedlings using the peeling inoculation method as previously described [8], with 16 replicates (individual plants) used in each treatment. Physiological saline solution as the negative control.

### 2.3 Trunk injection with EB and sampling procedure

The nematicide EB (cat no. 155569-91-8, Merck, Shanghai, China) was dissolved first in methanol, to prepare a stock solution (0.05 g/mL), then diluted to 4 mg/L with double-distilled water for the working solution, 4 mg/L methanol solution as control solution. In indoor experiments, 7 days post infection (dpi) after *P*. *massoniana* seedlings were inoculated with PWN, 20 seedlings inoculated with PWN were divided equally into two parts according to their growth, as previously described [25], one part injected with the treatment solution as the treatment group (PWN(+)_EB(+)) and the other part injected with the control solution as the control group (PWN(+)_EB(-)), and 20 seedlings inoculated with double distilled water were divided equally according to their growth One part was injected with the treatment solution as the treatment group (PWN(-)_EB(+)) and the other part with the control solution as the control group (PWN(-)_EB(-)); n = 10 for each treatment. Plant samples were harvested at 7 dpi. The collected samples were surface disinfected using ethanol solution, sodium hypochlorite solution and ethanol solution and then stored at -80°C.

A 2% EB solution (PD20110688, Syngenta, Shanghai, China) was used for the trunk injection in the field test. The injection dose was based on the diameter at breast height (DBH) of each plant (1 mL/cm). High-pressure trunk drilling and injection equipment (cat no. ZYJ15B, Greenman, Beijing, China) was used for each injection, by following a standard protocol (The depth of injection drilling is 5–10 cm, and a height of 1 m, with the drilled hole facing downwards at 45° angle to the horizontal plane). The number of PWN within the pine trees was categorically sampled according to section 2.1, with six randomly selected numbers of plants per treatment. From each tree, fresh shoots were selected along four directions (southeast, southwest, northeast, northwest), the collected samples are surface disinfected using ethanol solution, sodium hypochlorite solution and ethanol solution and then stored at -80°C.

### 2.4 Quantification of the relative number of PWN level

The amount of PWN in live plants was quantified by RT-qPCR (Real-time Quantitative Polymerase Chain Reaction). Fresh branches (with leaves) from four different directions of an adult plant in the field, and the whole plant of young seedling in the laboratory, were used for the PWN quantification. The total genomic DNA of each plant sample was extracted with the MoBio PowerPlant® Pro DNA Isolation Kit (cat no.12855-50, MoBio, USA), according to the manufacturer’s protocol. The DNA quantity and quality were measured on a NanoDrop 2000 photometer (Thermo Fisher Scientific, USA), and DNA integrity was determined by 1% agarose gel electrophoresis. The extracted DNA was stored at -80°C until further use. Quantitative PCRs were performed using PWN-specific primers, with non-specific primers (one random transcript from *P*. *massoniana*) as the internal control (S1 Table), and the Hieff^TM^ qPCR SYBR Green Master Mix (Low Rox Plus, cat no. 11202ES08, Yeasen, Shanghai, China) on a QuantStudio 6 Flex PCR system (ABI, MA, USA). The qPCR signals were normalized to those of the reference gene *PST* in pine trees by applying the 2^-ΔΔCT^ method [26]. Biological triplicates with technical triplicates were performed.

### 2.5 Quantification of plant defense genes

Total RNAs isolated from pine tree seedlings at 7 dpi with the Trizol reagent (cat no. 15596026, Invitrogen, CA, USA). Total RNA (1 mg) was reverse transcribed by the PrimeScript^TM^ RT reagent Kit with the gDNA Eraser (cat no. RR047A, Takara, Japan). The resistance genes were the same as those in our previous study [8], the internal reference gene is *PST*; the specific primers and reference genes are listed in S1 Table. RT-qPCR was conducted to quantify the gene expression levels, as described in section 2.5.

### 2.6 Quantification of reactive oxygen species in the host plant *Pinus massoniana*

The field was selected whether to inject EB cases with high, low, and no PWN content; six cases each of young leaves of *P*. *massoniana*. The EB-infected pine samples for the indoor experiments were harvested at 7 dpi. Collected pine needles were immediately placed in liquid nitrogen and stored in a -80° refrigerator. Then the quantity of ROS present (cat no. ROS-1-Y, Comin Biology, Suzhou, China) in the samples was measured by following standard protocols for the kits.

### 2.7 Metagenome sequencing and analysis

Concerning the microbiota within host pine trees, at 7 dpi the whole seedlings of *P*. *massoniana* (n = 5, with or without PWN) under the treatments were crushed in liquid nitrogen for their individual total DNA extractions, followed by metagenomic sequencing and analysis, as previously described [8, 27]. Total genomic DNA was extracted from each sample by using the MoBio PowerPlant® Pro DNA Isolation Kit (12855–50, MoBio, United States) as per the manufacturer’s protocol. The DNA quantity and quality were measured on a NanoDrop 2000 spectrophotometer (Thermo Fisher Scientific, United States). This DNA was then sheared to 300-bp fragments by a Covaris ultrasonic crusher. To prepare each sequencing library, those fragments were treated by end repair, A tailing, and ligation of Illumina compatible adapters. Next all DNA sequencing libraries were deep-sequenced on an Illumina HiSeq platform at the Allwegene Company (Beijing, China). After every run, the image analysis, base calling, and error estimation were carried out using Illumina Analysis Pipeline v2.6. Quality control of the raw data, including the removal of adapter sequence and low-quality reads, was performed using Trimmomatic. High-quality sequences were compared with NR database and classified into different taxonomic groups, using the DIAMOND tool [28]. Then MEGAHIT [29] was used to assemble the sequencing data, and the contigs were annotated with Prodigal software [30] to predict the open reading frames (ORFs). After that, CD-HIT software [31] constructed the non-redundant gene set. To compare the sequencing data with the non-redundant gene set, Bowtie [32] was used, after which the abundance of information of genes in the different samples was counted.

### 2.8 Metabolomic sequencing and analysis

Sample preparation went as described in section 2.3, with three technical replications used. The metabolites were then extracted from each sample by following a previously described protocol [33]. The Ultra High Performance Liquid Chromatography (UHPLC) separation was carried out using an A Dionex Ultimate 3000 RS UHPLC (Thermo Fisher Scientific, Waltham, MA, USA) equipped with an ACQUITY UPLC HSS T3 column (1.8 μm, 2.1×100 mm, 186009468, Waters, Milford, USA) by the Oebiotech Company (Shanghai, China). Set to a flow rate of 0.35 mL/min, the mobile phases were 0.1% formic acid in water (A) (A117-50, Thermo Fisher Scientific, Waltham, MA, USA) and 0.1% formic acid in acetonitrile (B) (A998-4, Thermo Fisher Scientific, Waltham, MA, USA). The column temperature was set to 45°C, while the auto-sampler temperature was set to 4°C, and the injection volume was 5 μL [34, 35]. Ensuing data were trimmed from different samples to distinguish the EB-induced metabolites. Next, commercial databases, including the Kyoto Encyclopedia of Genes and Genomes (KEGG; http://www.kegg.jp) and MetaboAnalyst (https://www.kegg.jp/) were utilized to search for ‘ metabolitepathways ‘ (https://www.genome.jp/kegg/pathway.html).

### 2.9 Insect diet quantification and gut metagenome analysis assay

The JPS adults were collected from an experimental population reared at the Fujian Agriculture and Forestry University, Fuzhou, China. All related experiments were conducted in a growth chamber (light: dark = 12 h: 12 h, 70% humidity, 25°C). The JPS that consumed *P*. *massoniana* injected with EB was the treatment group (EB(+)), and the JPS that consumed *P*. *massoniana* injected with double distilled water was the control samples (EB(-)).

The diet of JPS was quantified as the consumed area of *P*. *massoniana* bark by the insects. Healthy and vigorous JPS individuals were collected and starved for 24 h before the treatments commenced. A total of 12 JPS adults (6 males and 6 females) were used for each treatment. Fresh 2-year-old *P*. *massoniana* seedlings from the various treatments (injection of EB or double-distilled water) were fed to the JPS for 3 days, then the feeding area of the JPS was rubbed with tracing paper, and measured using grid coordinate paper.

All the JPS from the same treatment of each diet quantification assay were pooled into a single sample, crushed in liquid nitrogen, and divided into three equal aliquots for further analysis. Next, the activities of exo-β-1,4-glucanase/cellobiose hydrolase (cat no. G0533W, Gris Biology, Suzhou, China), endo-β-1,4-glucanase (cat no. G0534W, Gris Biology, Suzhou, China), and β-glucosidase (cat no. G0535W, Gris Biology, Suzhou, China) in each sample were measured by following standard protocols of corresponding kits. One-way analysis of variance (ANOVA; Tukey’s test) was implemented to determine the differences among the means of treatment groups.

Before preparing samples of the JPS intestinal microbiota, the surface of an individual adult was thoroughly rinsed with 70% ethanol and a sterile phosphate buffer. The JPS intestinal system was dissected under sterile conditions on sterile slides, using a pair of sterile tweezers and a sterile scalpel. The intestinal system (i.e., the midgut and hindgut) of each JPS were collected after its feeding on pine trees that had been inoculated with EB or the ethanol solution, and crushed in liquid nitrogen for total DNA extraction, the metagenomic sequencing and analysis according to the methods described above. Biological quintuplicates with technical triplicates were performed.

## 3. Results

### 3.1 EB eliminates the PWN and promotes the ROS accumulation in live host plants

**Fig 1:**
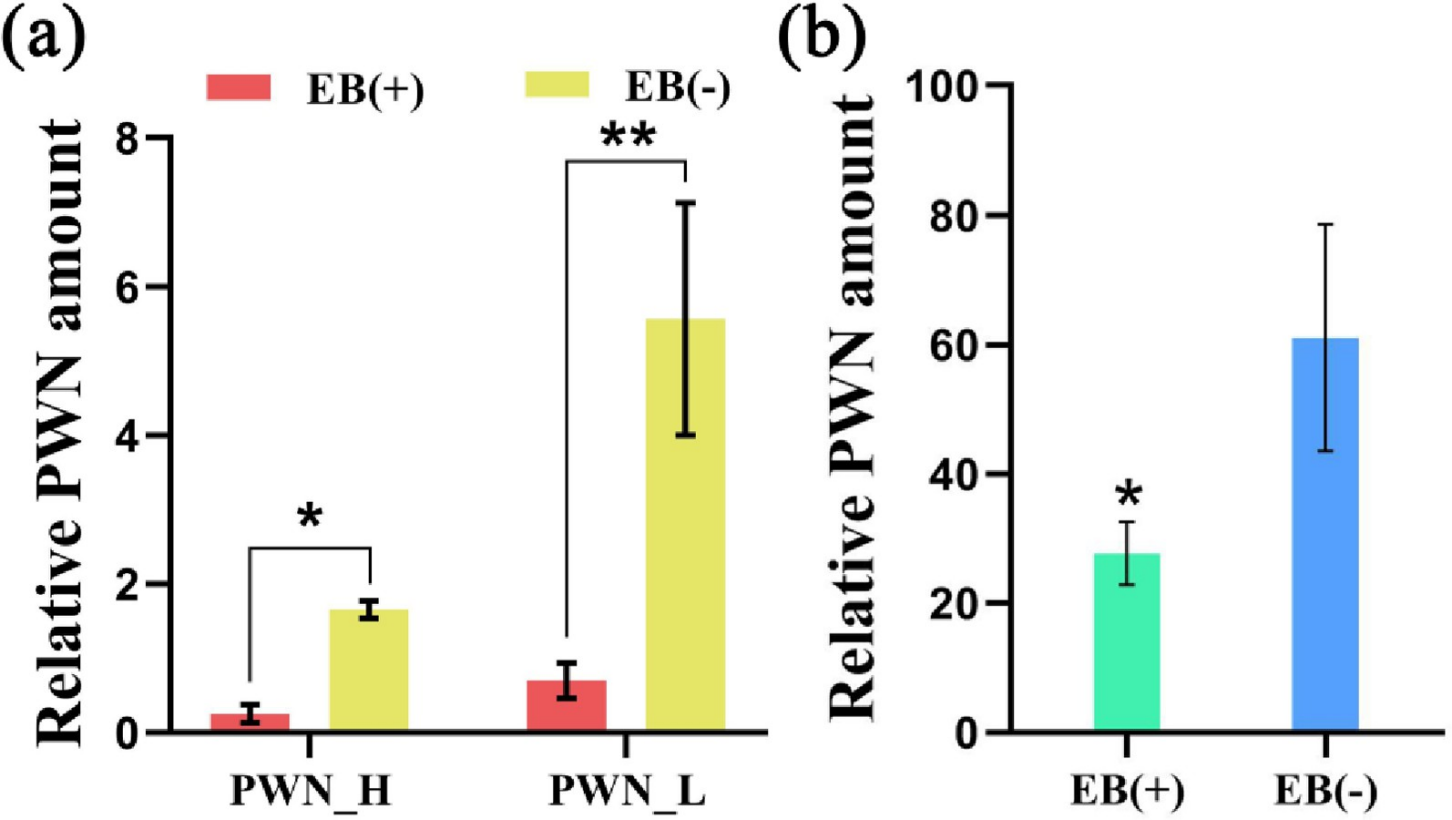
Impact of EB (emamectin benzoate) injection on the amount of PWN (pine wilt nematode) in the host plant *Pinus massoniana*. (a) The control efficacy of EB on the PWN in host trees carrying a high (PWN_H, PWN amount > 10000) and low (PWN_L, PWN amount < 6000) load of PWN in the field (a), and laboratory (b) were plotted according to the relative amount of PWN present. EB (+) and EB (-) represent injection with EB or a control chemical, respectively. The * represents a significant difference between samples, *P* ≤ 0.05, (a) with non-parametric tests (Kruskal-Wallis tests and Wilcoxon rank-sumtests), (b) based on a one-way ANOVA, with a multiple comparison analysis using Tukey’s test.

Two months after trunk injection with a 2% EB solution, we confirmed that EB was transported from the bottom of the trunk up to the leaves, by quantifying the residual abundance of EB in adult *P*. *massoniana* trees in the field (S2 Table). Additionally, the amounts of PWN were quantified in live adults and seedlings of *P*. *massoniana*. EB significantly decreased the amount of PWN in both the high (PWN_H, PWN amount > 10 000) and low (PWN_L, PWN amount < 6000) PWN-infected plants (*P* ≤ 0.01), respectively, reduced to 15.16% and 12.59% in the control group (Fig 1A). We also found that the amount of PWN in the *P*. *massoniana* seedlings was significantly reduced (*P* ≤ 0.05), and decreased to 45.36% of the control group (Fig 1B) after the EB injection. In addition, PWN with low carrying capacity cannot kill *P*. *massoniana* within 56 days (S2 Fig).

**Fig 2:**
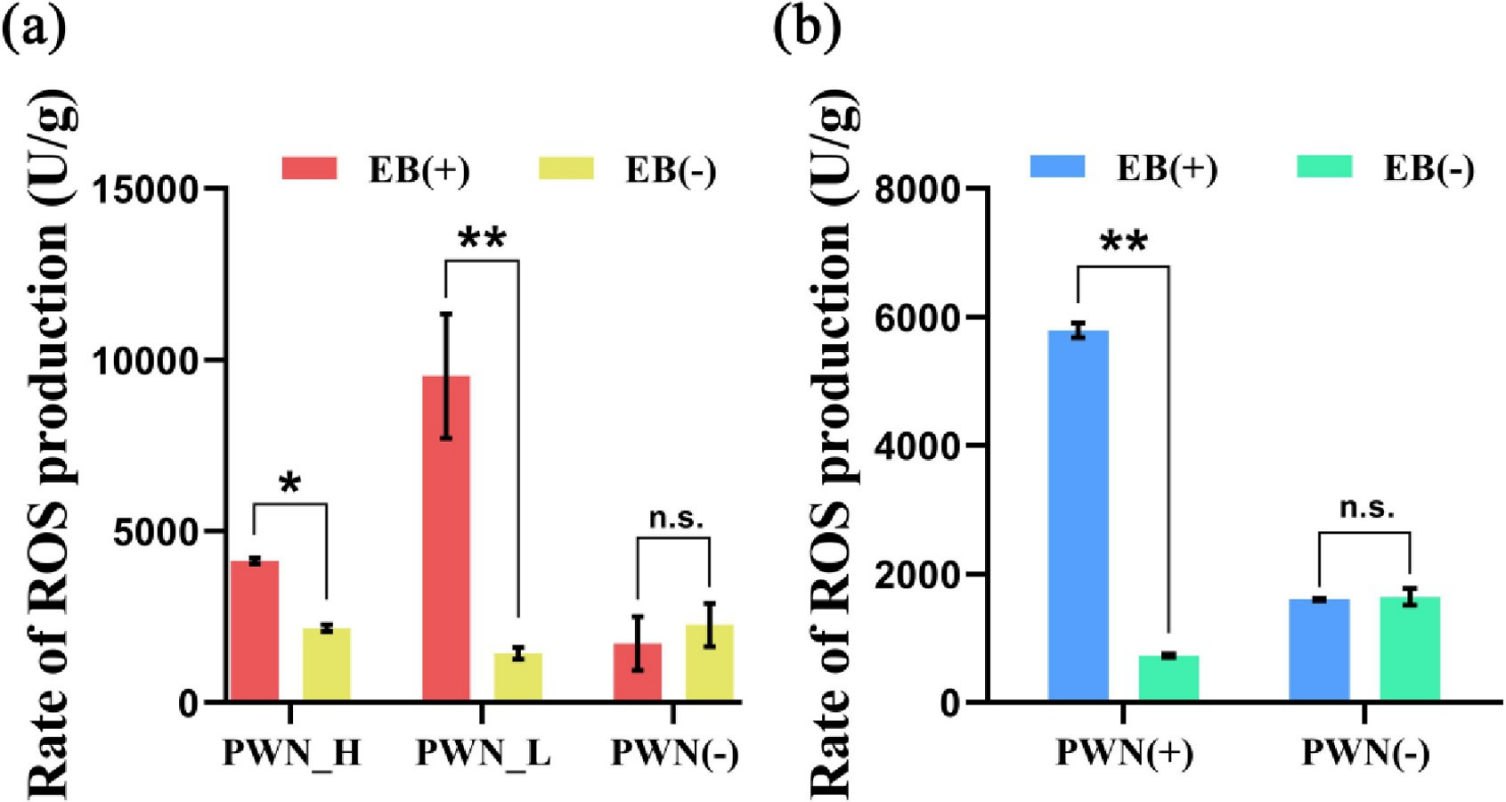
Injection of EB (emamectin benzoate) promotes ROS accumulation in the host plant *Pinus massoniana*. The ROS level was quantified in field (a) and indoor seedling (b) samples. EB (+) and EB (-) respectively denote the injection of EB or a control chemical. PWN_H and PWN_L represent high and low amounts of PWN carriers in the field; PWN (+) or PWN (-) indicate whether the host plant carries PWN or not, respectively. The symbol * indicates a significant difference between samples at *P* < 0.05, the symbol ** indicates a highly significant difference between samples at *P* < 0.01, while “n.s.” indicates no significant difference between samples, with non-parametric tests (Kruskal-Wallis tests and Wilcoxon rank-sumtests).

Furthermore, we also measured the accumulation of ROS in adults and seedlings of *P*. *massoniana*. We found that the injection of EB significantly (*P* ≤ 0.01) increased the rate of ROS production in PWN_H and PWN_L plants by 1.9-fold and 6.6-fold, respectively. In the sample without PWN, no significant difference was evident (Fig 2A). In the presence of the PWN, ROS production was significantly augmented by EB (*P* < 0.01), but no significant difference was detected in the PWN-free samples (Fig 2B).

### 3.2 Injection of EB enhances resistance to PWN in living host plants

**Fig 3:**
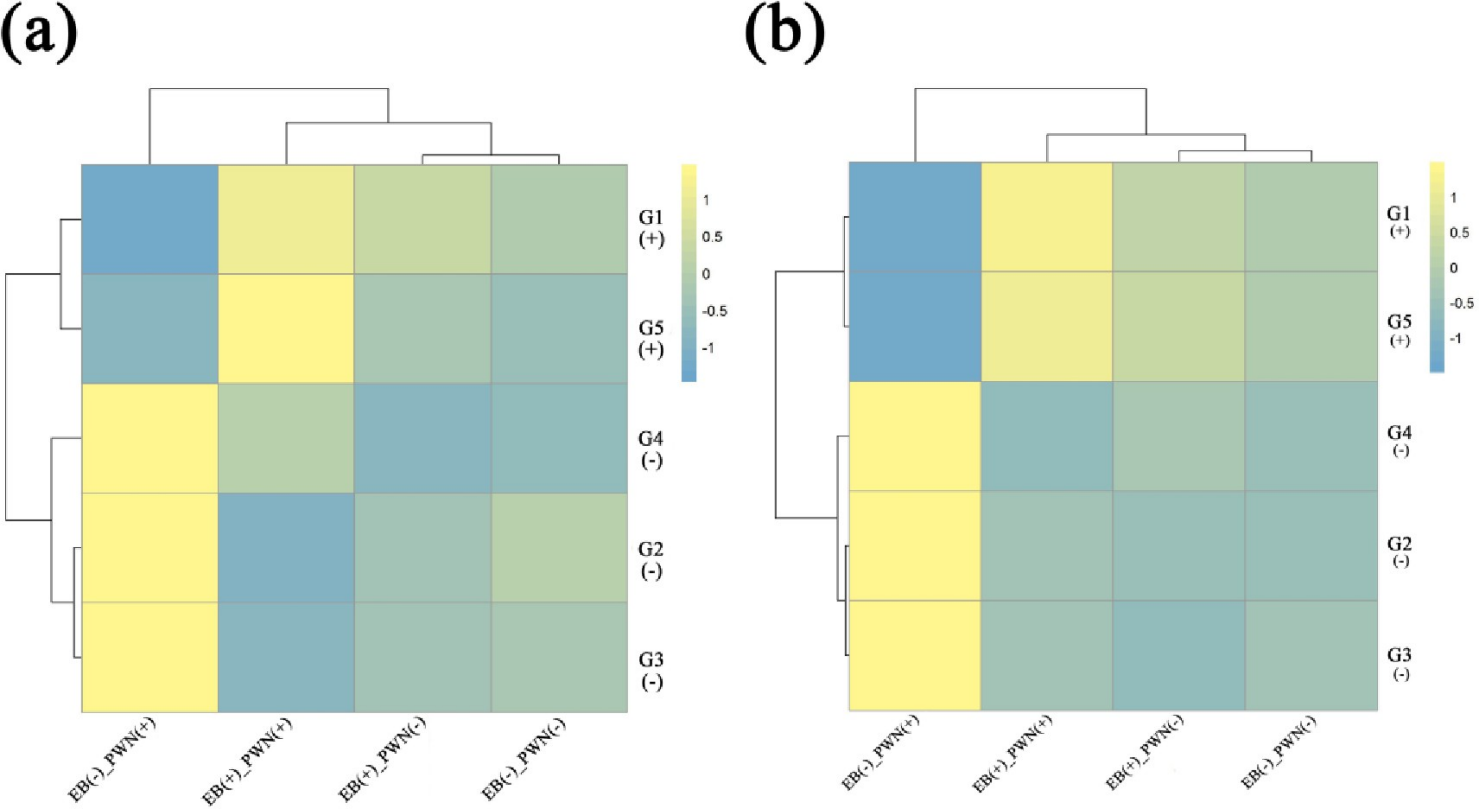
Impact of EB (emamectin benzoate) injection on the defense genes of the host plant *Pinus massoniana*. The PWN resistance genes of pine trees were quantified in the field (a) and seedlings (b); *G1*–*G5* denotes gene *c60547*.*graph_c0*, *c64867*.*graph_c0*, *c68789*.*graph_c0*, *c81022*.*graph_c0*, and *c82953*.*graph_c0*, respectively. PWN (+) represents the host plants carrying the PWN, and PWN (-) those not.

Furthermore, we quantified the resistance genes, the microbiota, and the metabolome of *P*. *massoniana* plants. We found that EB up-regulated the expression levels of two positive-related resistance genes, *G1* and *G5*, significantly (*P* < 0.05) in both adults and seedlings by 1.6-fold and 8139-fold, respectively. However, the expression levels of the negative-related resistance genes *G2*–*G4* were significantly decreased (*P* < 0.05) by 27-, 11-, and 3-fold, respectively. EB did not change the expression level of any resistance genes in the absence of the PWN (Fig 3).

**Fig 4:**
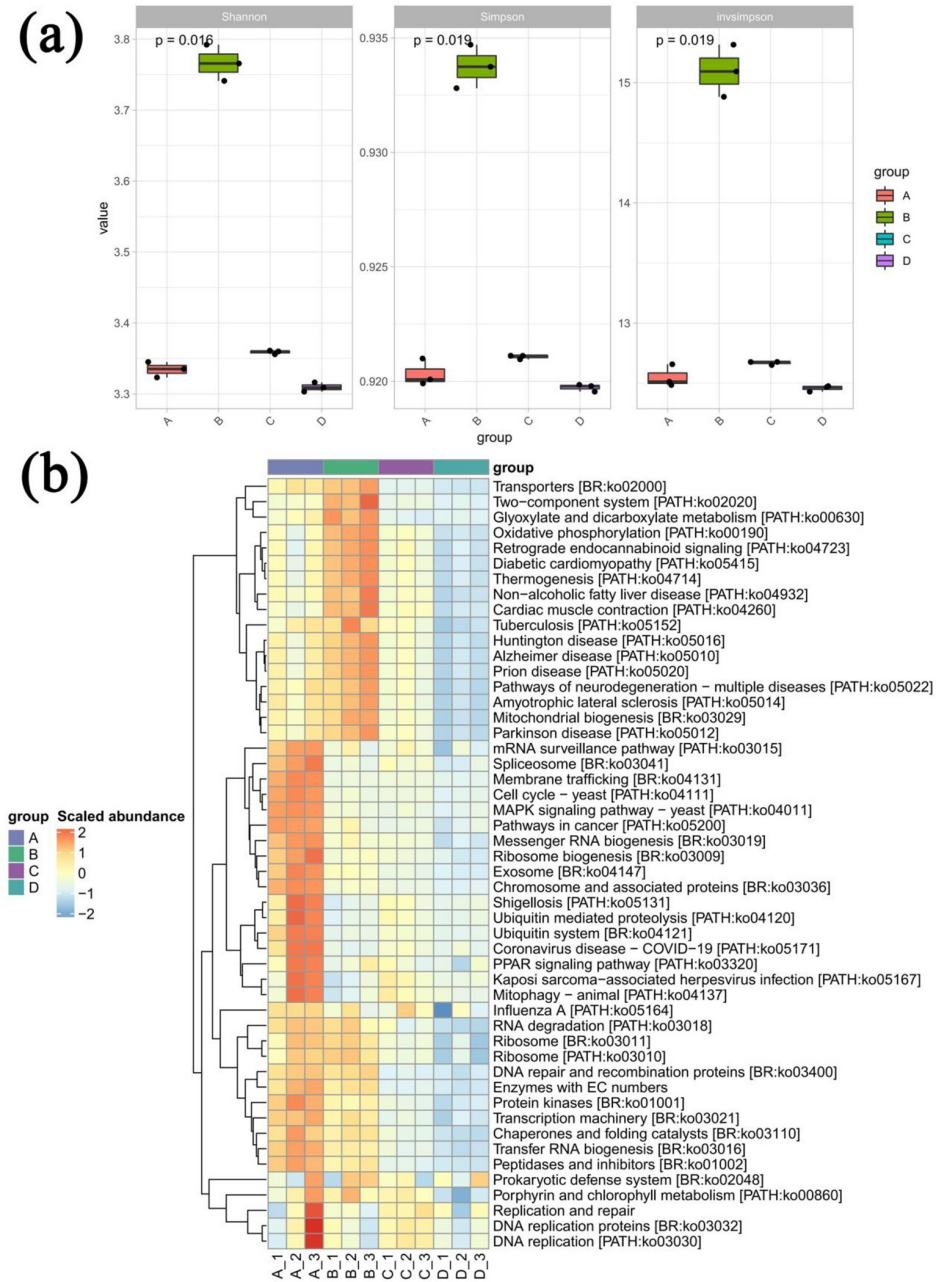
Impact of EB (emamectin benzoate) injection on the symbiotic microbiota of the host plant *Pinus massoniana*. The impact of EB on symbiotic microbiota of *P*. *massoniana* seedlings in terms of alpha diversity (a) and KEGG functional abundance of microbes present in differing proportions (b). Samples A, B, C, and D represent the seedlings containing PWN and EB, EB alone, PWN and a controlled chemical, and the controlled chemical alone, respectively, with non-parametric tests (Kruskal-Wallis tests and Wilcoxon rank-sum tests).

All of the associated bacteria belonged to 1 kingdom, 44 phyla, 817 classes, 162 orders, 317 families, 603 genera, and 1423 species. The associated fungi belonged to 1 kingdom, 7 phyla, 28 classes, 66 orders, 152 families, 218 genera, and 344 species. The associated viruses belonged to 1 kingdom, 6 phyla, 7 classes, 7 orders, 8 families, 13 genera, and 12 species. Microbial community diversity was measured using several alpha diversity indexes. Of all samples, those injected with EB-free PWN had the highest Shannon, Simpson, and Invsimpson indexes (Fig 4A). In addition, in the presence of PWN the EB injection significantly (*P* < 0.05) increased the Shannon diversity index of the microbial community, whereas in the absence of PWN the EB injection significantly (*P* < 0.01) increased the Shannon, Simpson, and Invsimpson indexes (S3 Fig). The KEGG functional abundance heat map showed that the P samples injected with EB had significantly higher microbial functional abundance in the Transporters, Glyoxylate and dicarboxylate metabolism, Oxidative phosphorylation, Mitochondrial biogenesis Peptidases and inhibitors, Prokaryotic defense system, and Porphyrin and chlorophyll metabolism were significantly more abundant than controls (Fig 4B).

**Fig 5:**
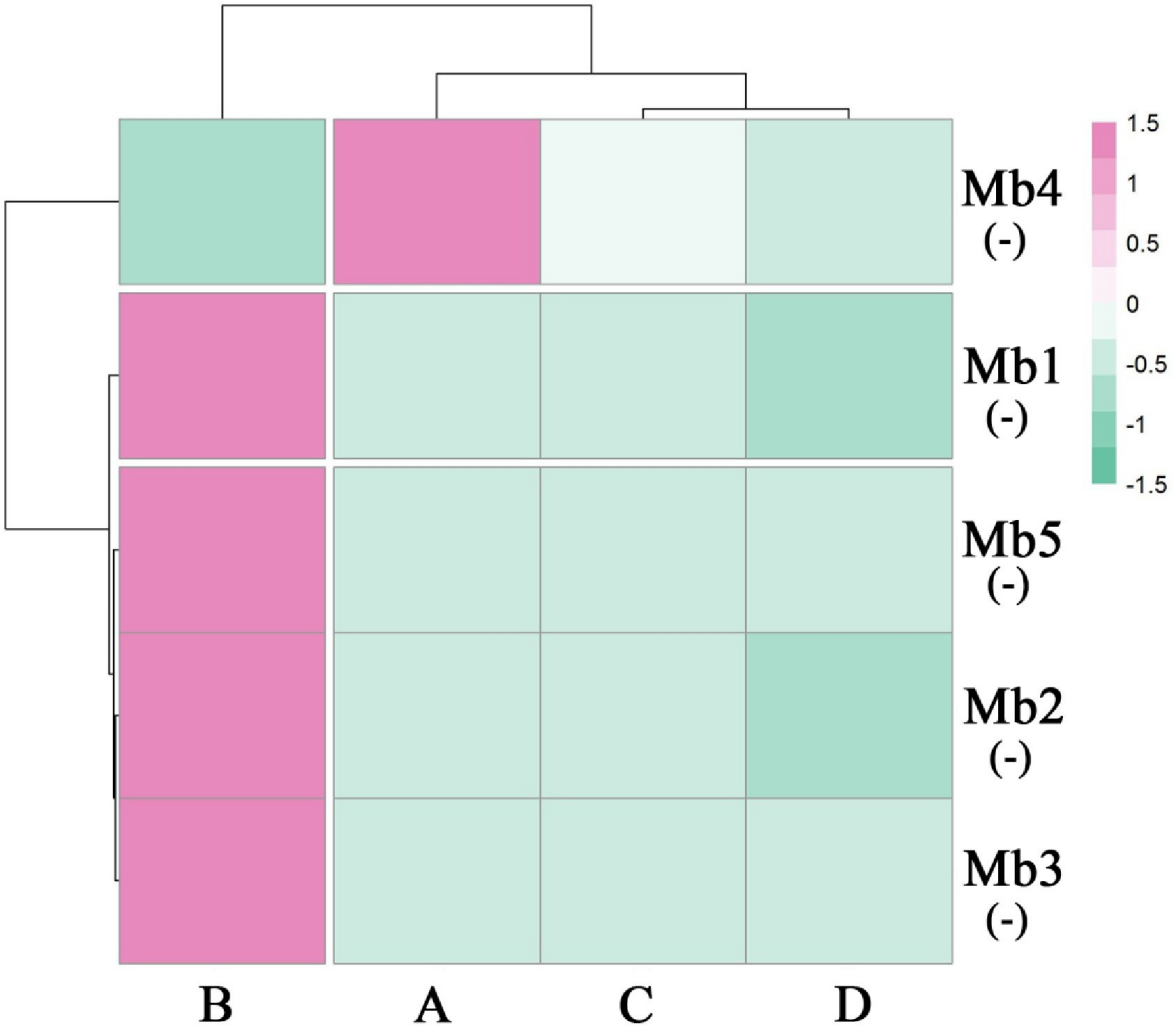
The impact of EB on symbiotic microbiota of *P*. *massoniana* seedlings in terms of the richness of ROS accumulation-related microbes in different seedlings plotted. Samples A, B, C, and D represent the seedlings containing both PWN and EB, EB alone, PWN and a controlled chemical, and the controlled chemical alone, respectively, Mb1–Mb5 represent *Cladophialophora*, *Penicillium*, *Trichoderma*, *Achromobacter*, and *Chitinophaga*. The symbol “(+)” or “(-)” following the name of a microbe indicates its stimulation or suppression related to ROS accumulation.

**Fig 6:**
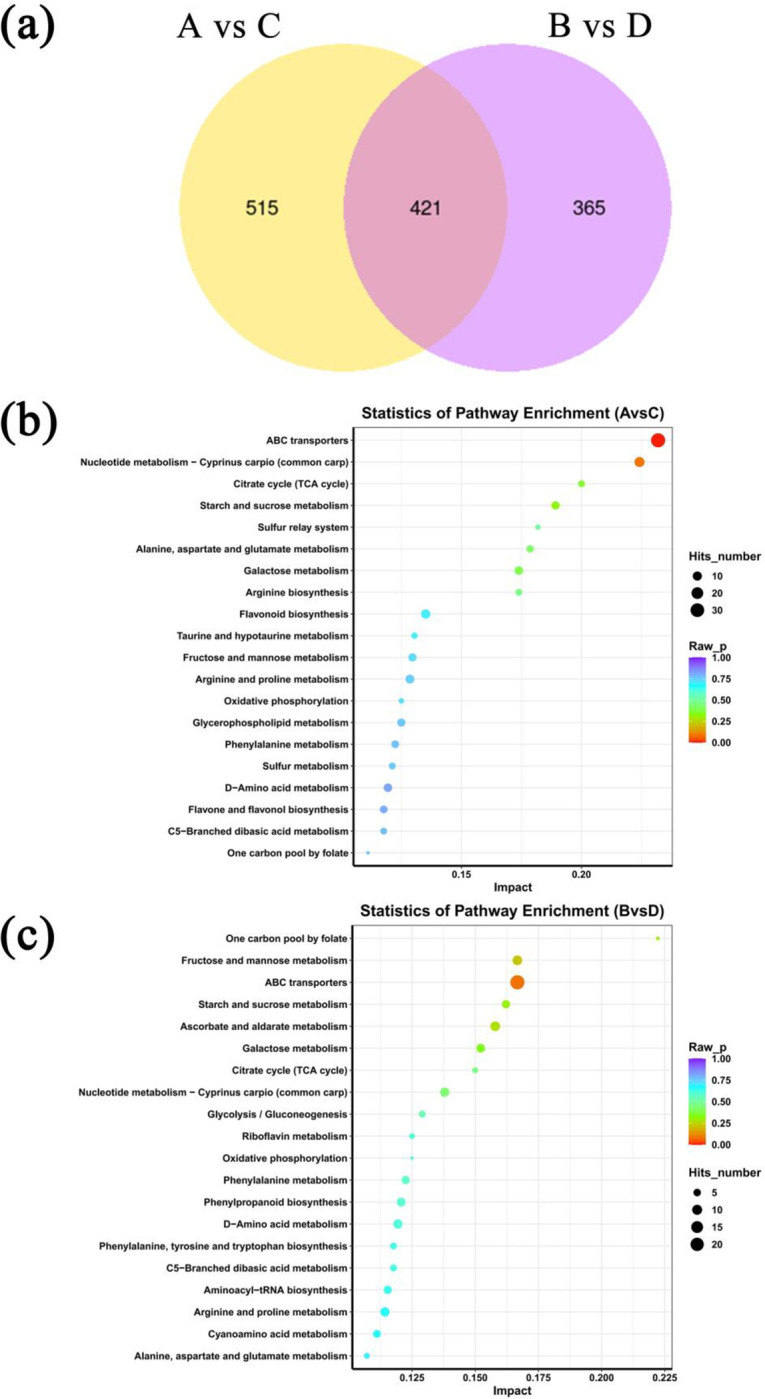
Impact of EB (emamectin benzoate) injection on the metabolites of the host plant *Pinus massoniana*. The impact of EB on the metabolites of *P*. *massoniana* seedlings in terms of the number of metabolites (a), the functions of differentially induced metabolites (b, c) and the richness of ROS accumulation-related metabolites (d) in different seedlings plotted. Samples A, B, C, and D represent those seedlings containing both PWN and EB, EB alone, PWN and a controlled chemical, and the controlled chemical alone, respectively, Mt1–Mt7 represent tyramine, 1,2-di-(9Z-octadecenoyl)-sn-glycero-3-phosphocholine, 1-hexadecanoyl-2-octadecadienoyl-sn-glycero-3-phosphocholin, linalool oxide, 3alpha,6alpha-mannotriose, cellobiose, and maltotriose, respectively. The symbol “(+)” or “(-)” following the name of a metabolite indicates its stimulation or suppression related to ROS accumulation.

Our previous work identified microbes that were highly correlated with ROS accumulation in the host plant *P*. *massoniana* [8], so here we checked the richness of these microbes in the experimental hosts. In the presence of the PWN, the richness of those microbes (*Cladophialophora*, *Penicillium*, *Trichoderma* and *Chitinophaga*) inversely related to ROS accumulation was significantly decreased after EB injection (*P* < 0.05) vis-à-vis the control, except for *Achromobacter* (Fig 5). However, in the PWN-free samples, the EB injection did not affect the richness of these microbes (Fig 5).Overall, 1964 metabolic substances were induced in the various pine tree seedling samples. To further characterize the effects of the EB injection on host metabolites, we mapped the metabolic profiles of the PWN carriers and non-carriers. Of these, A (seedlings containing both PWN and EB) vs. C (seedlings containing PWN and a controlled chemical) is the effect of EB on plants injected with PWN, and B (seedlings containing EB only) vs. C is the effect of EB on plants in the absence of PWN. For both comparisons, a total of 421 common induced metabolites were present, with 515 and 365 specifically induced metabolites, respectively (Fig 6A). A principal component analysis (PCA) of the samples (including the quality control samples) were done to preliminarily determine the overall metabolic differences and variability between samples. In the presence of the PWN, the contribution of the first principal component (PC1) was 59.7%, while that of the second (PC2) was 30%; hence 89.7% of the variance was explained by both components. This PCA showed that EB-injected samples could be clearly distinguished from non-EB-injected samples (S4 Fig). In the absence of the PWN, the contribution of PC1 was 61.1%, while that of the PC2 was 28.4%, totaling 89.5%. This PCA showed that EB-injected samples could be clearly distinguished from non-EB-injected samples (S5 Fig). Similarly, a clustering heat map analysis revealed that the composition of induced metabolites was similar within groups. The metabolites induced by EB injection differed from those in the other samples (S6 Fig). Moreover, the KEGG enrichment analysis of the differential metabolites indicated that, in the presence of the PWN, the differential metabolites were primarily enriched for ABC transporters and the nucleotide metabolism-*Cyprinus Carpio* (common carp) pathway (Fig 6B). The differential metabolites were mainly enriched in ABC transporters and the fructose and mannose metabolism pathway in the absence of PWN (Fig 6C). A multivariate analysis of the variable importance projection (VIP) of the Orthogonal Partial Least Squares-Discriminant Analysis (OPLS-DA) model facilitated the initial screening of metabolites that most differed between different species. We also combined the *P*-value and fold-change (i.e., the differential fold-change value) of the univariate analysis to further screen differentially induced metabolites between different treatments. The VIP values of different metabolites were used to rank the top 20 metabolites, for which the differences between different treatments were pronounced (S7 Fig).

**Fig 7:**
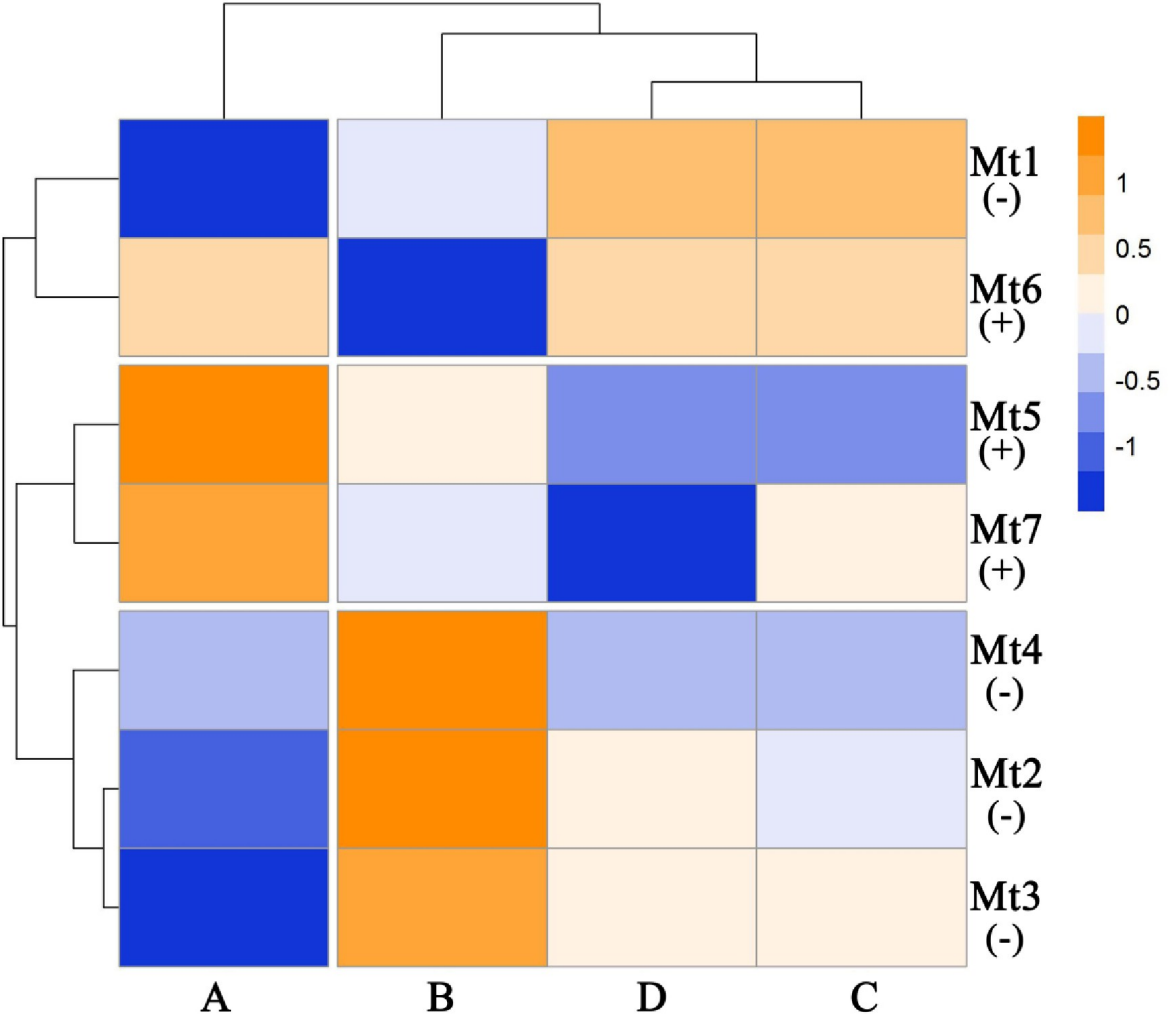
The impact of EB on the metabolites of *P*. *massoniana* seedlings in terms of the number of the richness of ROS accumulation-related metabolites in different seedlings plotted. Samples A, B, C, and D represent those seedlings containing both PWN and EB, EB alone, PWN and a controlled chemical, and the controlled chemical alone, respectively, Mt1–Mt7 represent tyramine, 1,2-di-(9Z-octadecenoyl)-sn-glycero-3-phosphocholine, 1-hexadecanoyl-2-octadecadienoyl-sn-glycero-3-phosphocholin, linalool oxide, 3alpha,6alpha-mannotriose, cellobiose, and maltotriose, respectively. The symbol “(+)” or “(-)” following the name of a metabolite indicates its stimulation or suppression related to ROS accumulation.

Our previous work identified metabolites that were highly correlated with ROS accumulation in the host plant *P*. *massoniana* [8] (Cai et al. 2022), so here we checked the richness of these metabolites in the experimental hosts. In the presence of the PWN, the related metabolites tyramine, 1,2-di-(9Z-octadecenoyl)-sn-glycero-3-phosphocholine, 1-hexadecanoyl-2-octadecadienoyl-sn-glycero-3-phosphocholin, and linalool oxide, all of which were negatively correlated with ROS accumulation, decreased significantly. However, the positively correlated metabolites (i.e., cellobiose and maltotriose) did increase significantly; the EB injection did not affect the contents of these metabolites in the PWN-free samples (Fig 7).

### 3.3 EB affects the diet of JPS

**Fig 8:**
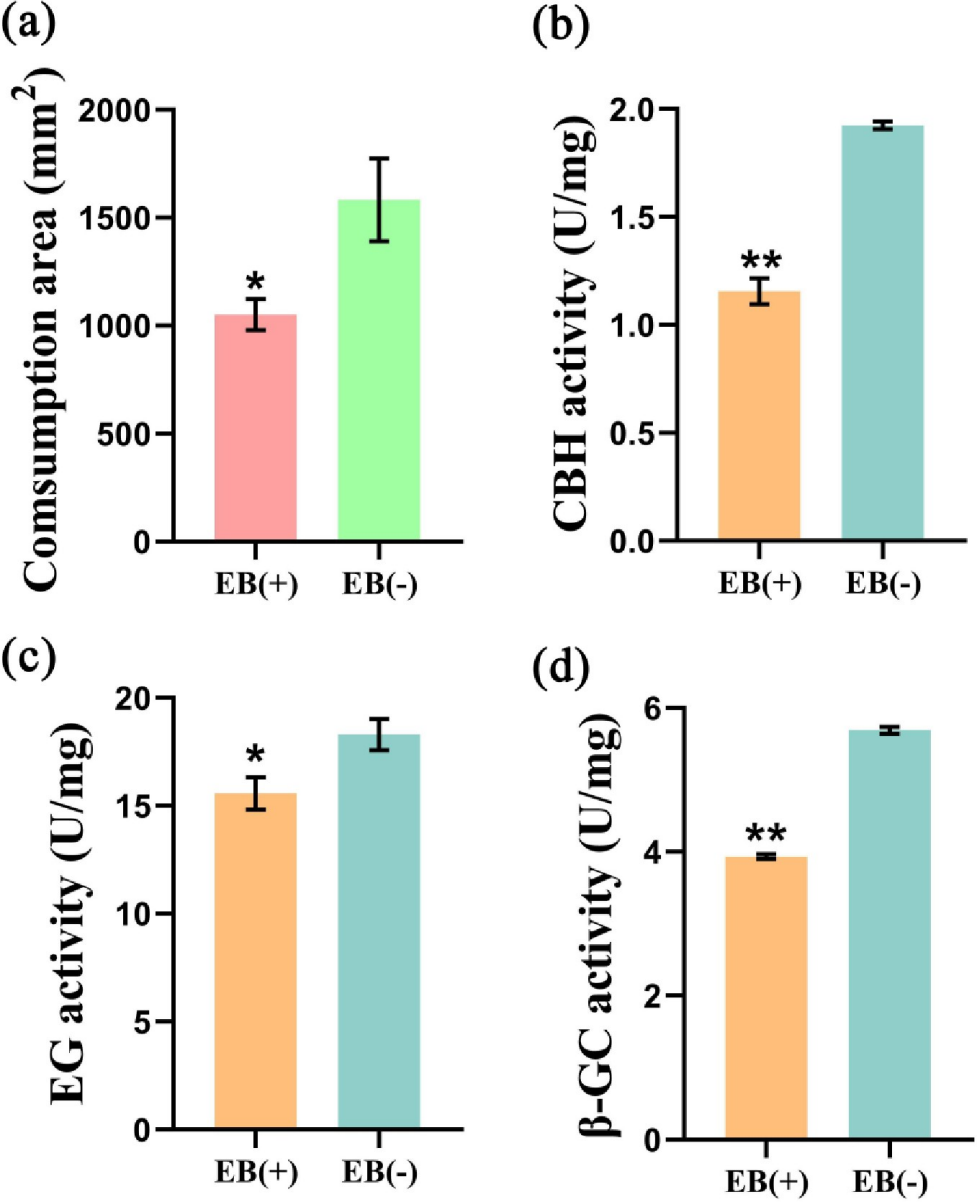
EB (emamectin benzoate) affects the diet of JPS (Japanese pine sawyer). Adults of JPS fed on different seedlings, and the amount of seedling tissue consumed (a) was quantified, along with CBH (Exo-β-1,4-glucanase/cellobiose hydrolase, b), endo-β-1,4-glucanase (c), and β-glucosidase (d) activity of JPS. EB (+) or EB (-) denotes seedlings injected with EB or a controlled chemical, respectively. The symbols * and ** indicate significant differences between samples at *P* < 0.05 and *P* < 0.01, respectively, based on a one-way ANOVA, with a multiple comparison analysis using Tukey’s test.

**Fig 9:**
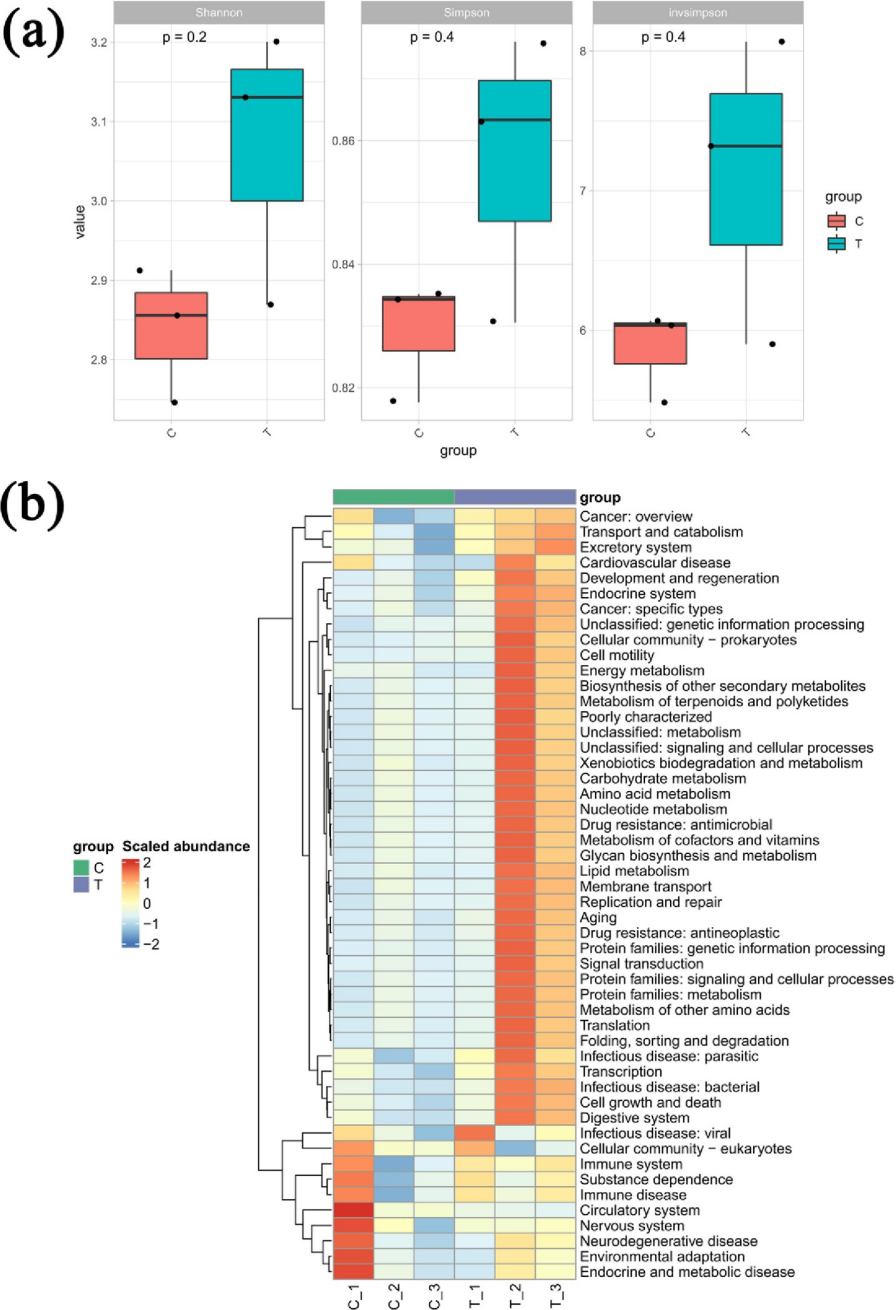
EB (emamectin benzoate) affects the gut microbiota of JPS (Japanese pine sawyer) through its diet. The gut microbiota of different JPS samples were compared in terms of their diversity (a) and function of differentially present microbes (b). The symbols “T” and “C” denote JPS feeding on seedlings containing EB or a controlled chemical, respectively. Significant differences between samples are based on a one-way ANOVA, with a multiple comparison analysis using Tukey’s test.

Furthermore, we also observed whether feeding on EB-treated P. massoniana could adversely affect the development of JPS and found that the consumption of *P*. *massoniana* seedlings by JPS was significantly (*P* < 0.05) reduced by EB (Fig 8A). To further explore the biochemical mechanism by which EB impacted the JPS diet, we quantified the activity of exo-β-1,4-glucanase/cellobiose hydrolase, endo-β-1,4-glucanase and β-glucosidase—which are positively related to the cellulase digestion in JPS—and all were significantly (*P* < 0.05) decreased by EB (Fig 8B–8D). Gut microbiota are crucial to food digestion and the development of insects, so we also investigated the gut microbial community of JPS via metagenomic sequencing. This revealed that eating EB-injected seedlings increased the gut microbial community diversity of JPS (Fig 9A). A PCA showed that the contribution of PC1 was 93.77%, whereas that of PC2 was very small (3.34%), summing to 97.11%, thus indicating that differences between different samples could be clearly distinguished (S8 Fig). Further, based on the functional annotations and abundance of differential microbiota between samples, we found that EB increased the richness of those genes that function as transcription factors and bacterial motility proteins, as well as in flagellar assembly, astarch and sucrose metabolism, the phosphotransferase system (PTS), pyruvate metabolism, and amino sugar and sugar nucleotide metabolism (Fig 9B).

## 4. Discussion

Our research demonstrates that EB injection significantly suppresses the amount of PWN in live adult and young *P*. *massoniana*. We also find that *P*. *massoniana* injections of EB can limit JPS feeding and affect the growth and development of JPS. These results successfully document the impact of EB on the resistant response of the *P*. *massoniana* against PWN and upon the latter’s insect vector (JPS).

In the field test, a 2% EB solution was delivered into the trunk of the host plant using a high-pressure injecting machine. This method has been proven to work well in several pine trees species (*P*. *pinaster*, *P*. *hunbergia*, and *P*.*densiflora*) because it can successfully deliver EB from bottom of the trunk to the twig parts [36, 37]. In the present work, we confirmed that EB could be transported by at least 25 m upward in a *P*. *massoniana* tree within 1 month by that system when applied in the field. This result is crucial for preventing and mitigating outbreaks of PWD in China given that *P*. *massoniana* is the important host of PWN and dominates China’s pine communities [38]. However, the EB concentration in the twig of the host plant (*P*. *massoniana*) was only about 2 mg/L after 1 month, which is half of the known Lethal Concentration 95 (LC95) of EB for PWN [20], and it was relatively lower compared to other pine tree species, perhaps due to the higher resin pressure of *P*. *massoniana* than other pine spp. thought it may be rescued by increasing the dosage or total injection amount used [20, 36, 37]. Nevertheless, the 2% EB solution is able to significantly reduce the PWN population in a live *P*. *massoniana* trees in the field. We believe this may come from enhanced PWN resistance (like ROS accumulation) of *P*. *massoniana* induced by EB. Thus, the potential of EB as a long-term nematicide in other host species warrants further study.

After the injecting EB, the genetic resistance of the host plant against the PWN improved substantially in a PWN-dependent manner, as did the composition of the symbiotic microbial community in the host. In the presence of PWN, EB increased the abundance of microorganisms that promote growth and development and control plant diseases in *P*. *massoniana*, and increased the accumulation of metabolites associated with growth and development and stress responses in *P*. *massoniana*, which in turn increased the ability of *P*. *massoniana* to resist PWN [39, 40], these changes may be ascribed to PWN being the direct target of EB [14, 19]. Additionally, the presence of EB highly suppressed the PWN population in a rather short period, which may affect the feedback loop of the host plant response to a nematode invasion [8, 22, 41, 42]. Unfortunately, we could not collect sufficient evidence to prove this hypothesis, and it therefore requires further research. Surprisingly, from UAV spectral data, we did find that injection of EB greatly ameliorated the symptoms caused by PWD [7, 36], by reversing the reduction in chlorophyll content and tree twig moisture in the host plant (*P*. *massoniana*) caused by the PWN infection from (S9 Fig). This result suggests the effect of EB on the host plant could change over a longer period.

This work confirms that EB can improve the ability of *P*. *massoniana* to resist PWN by increasing the level of ROS. This phenomenon greatly favors the control of PWN in the forestry stands. For the host plant, the ROS accumulation is temporarily toxic, but the tolerance of ROS differs among plant species, and ROS accumulation is a common side effect of pesticides yet often within the bounds of plant tolerance [43, 44]. Unfortunately, the tolerance of *P*. *massoniana* to ROS accumulation is still unstudied. Our planned future work will explore the dynamics of EB-induced ROS accumulation in *P*. *massoniana*, as well as the time-course of damage caused by ROS to infected *P*. *massoniana* hosts.

There is much evidence indicating that EB can damage insects that consume it, including several species in *Monochamus* (Coleoptera: Cerambycidae), by disturbing the insect’s appetence for feeding under natural conditions [19, 24, 36, 37, 45]. Our work further proved that, similar to *P*. *densiflora* [45], feeding on the *P*. *massoniana* can also significantly reducing the feeding appetence of JPS. Meanwhile, based on the biochemical and gut microbial data of JPS, we show that EB can significantly suppress cellulose digestion as well as altering both sugar metabolism and the phosphotransferase system; collectively, this is likely to exert a negative impact on the life-cycle development of JPS [46–48]. Moreover, we found that EB increases the expression level of genes that function as transcription factors in the gut of JPS. This result is consistent with previous work showing that EB can modulate the transcription in its targets [14].

This study demonstrates the process of EB effects on the PWN and between the host *P*. *massoniana*, however, the tolerance of the host *P*. *massoniana* to ROS after EB injection is ambiguous and needs further study. In addition, it was found that EB could affect the growth and development of JPS and block the transmission pathway of PWN, thus achieving the purpose of controlling pine wilt disease. Our research team is dedicated to devising a suitably effective approach based on this promising idea. This approach will highly reduce the overall cost and labor for PWD control, thereby enhancing its efficiency and enabling full control of PWD in pine stands in the near future.

## Supporting information

S1 Table Specific primers. (XLSX)

S2 Table EB amount in adult pine tree post-injection. (XLSX)

S1 Fig Aerial view of the study area. (DOCX)

S2 Fig Line chart of survival rate (%) of *Pinus massoniana* infected with PWN under different treatments. (DOCX)

S3 Fig Effects of injection of EB (emamectin benzoate) on microbiote diversity of *Pinus massoniana*. (DOCX)

S4 Fig Effects of EB injection without PWN on PCA analysis of plant metabolism. (DOCX)

S5 Fig Principal component analysis of EB-induced plant metabolism in the presence of PWN. (DOCX)

S6 Fig Effects of PWN injection on metabolites of host plants. (DOCX)

S7 Fig Effect of PWN injection on VIP of host plant. (DOCX)

S8 Fig Principal component analysis of JPS feeding on intestinal microbes with or without EB injection. (DOCX)

S9 Fig Effect of EB on chlorophyll content and water content of *Pinus massoniana*. (DOCX)
