# Supplementary material for: The nematicide emamectin benzoate increases ROS accumulation in Pinus massoniana and poison Monochamus alternatus
Source: PLoS One. 2023 Dec 21;18(12):e0295945. doi: 10.1371/journal.pone.0295945 (PMC10735008; doi:10.1371/journal.pone.0295945)
Supplement: S1 Fig — (DOCX) [file pone.0295945.s003.docx]

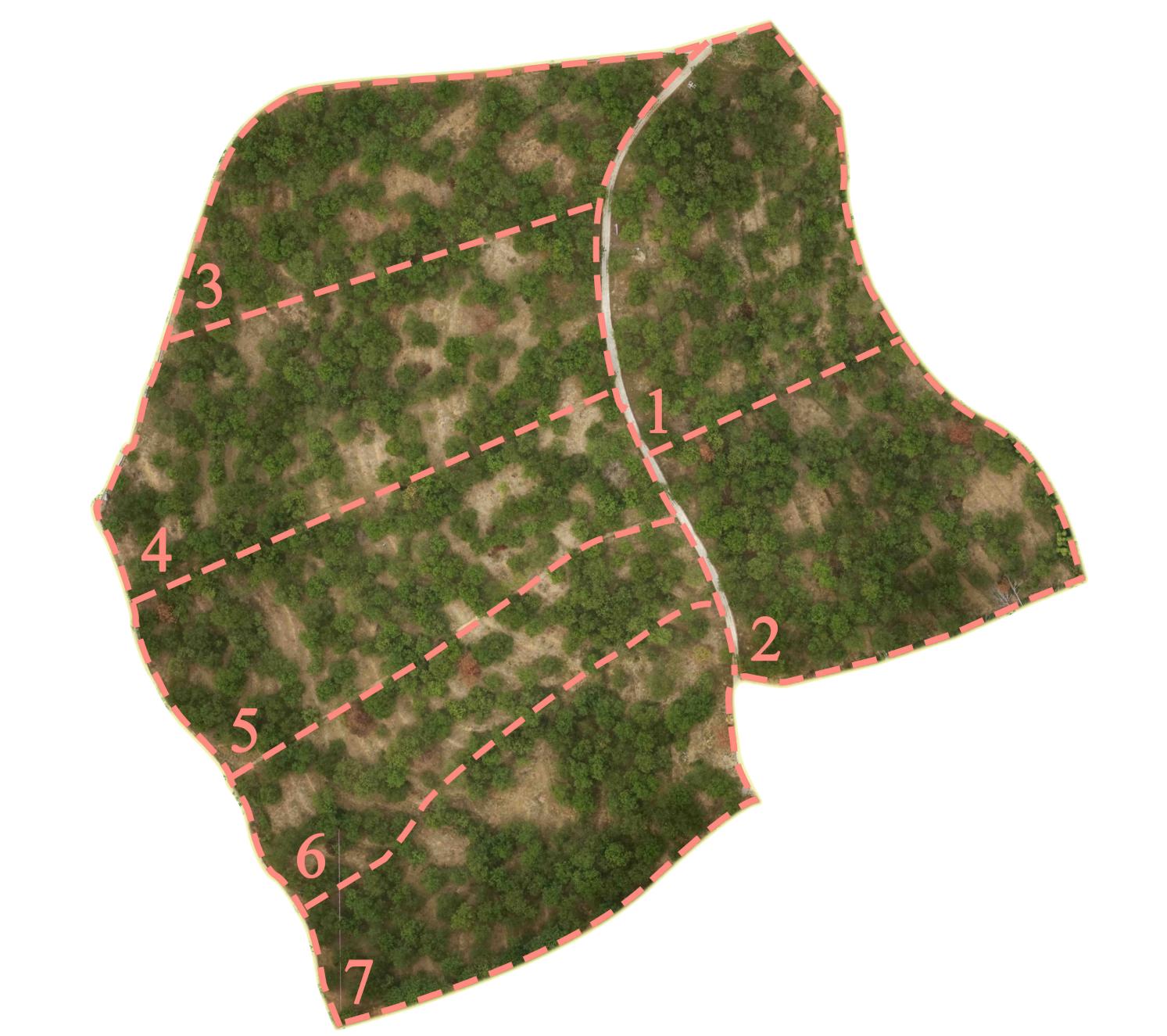


**Supplementary Figure S1. Aerial view of the study area*.***

Numbered as 1 ~ 7, in which plot 1~ 5 and 7 were served as treatment area and plot 6 were served as control.
