# Supplementary material for: The nematicide emamectin benzoate increases ROS accumulation in Pinus massoniana and poison Monochamus alternatus
Source: PLoS One. 2023 Dec 21;18(12):e0295945. doi: 10.1371/journal.pone.0295945 (PMC10735008; doi:10.1371/journal.pone.0295945)
Supplement: S2 Fig — (DOCX) [file pone.0295945.s004.docx]

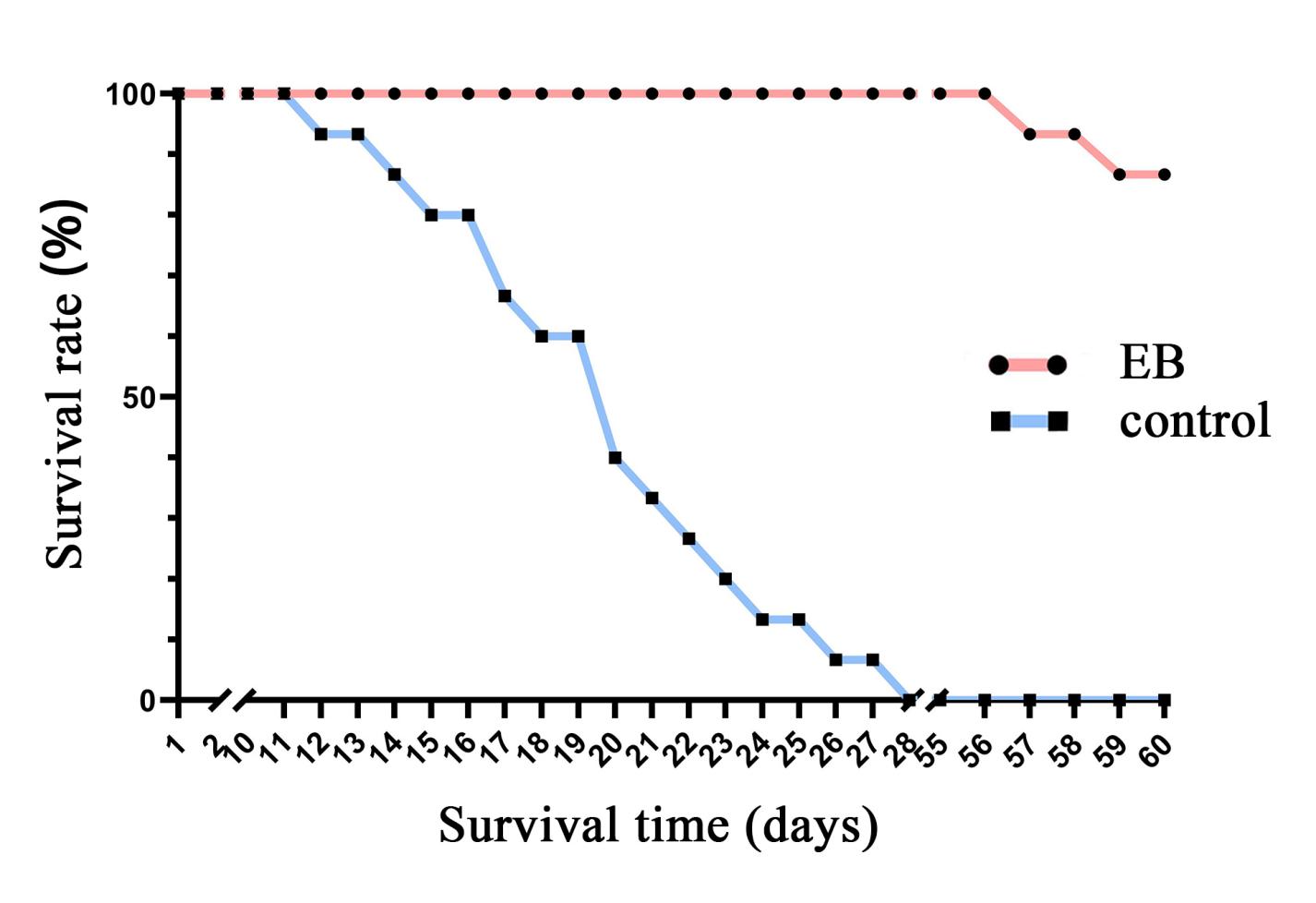


**Supplementary Figure S2. Line chart of survival rate (%) of *Pinus massoniana* infected with PWN under different treatments.**

We randomly selected 30 2-year-old P. massoniana seedlings that were in good health and not infected with PWN. After inoculating with PWN, half of the seedlings (15 individuals) were injected with 1 ml 4ppm EB solution as a test group and the other half were injected with methanol solution (1ml 4 ppm) as a control group. The survival ratio of P. massoniana were counted till 60 days post injection.
