# Supplementary material for: The nematicide emamectin benzoate increases ROS accumulation in Pinus massoniana and poison Monochamus alternatus
Source: PLoS One. 2023 Dec 21;18(12):e0295945. doi: 10.1371/journal.pone.0295945 (PMC10735008; doi:10.1371/journal.pone.0295945)
Supplement: S3 Fig — (DOCX) [file pone.0295945.s005.docx]

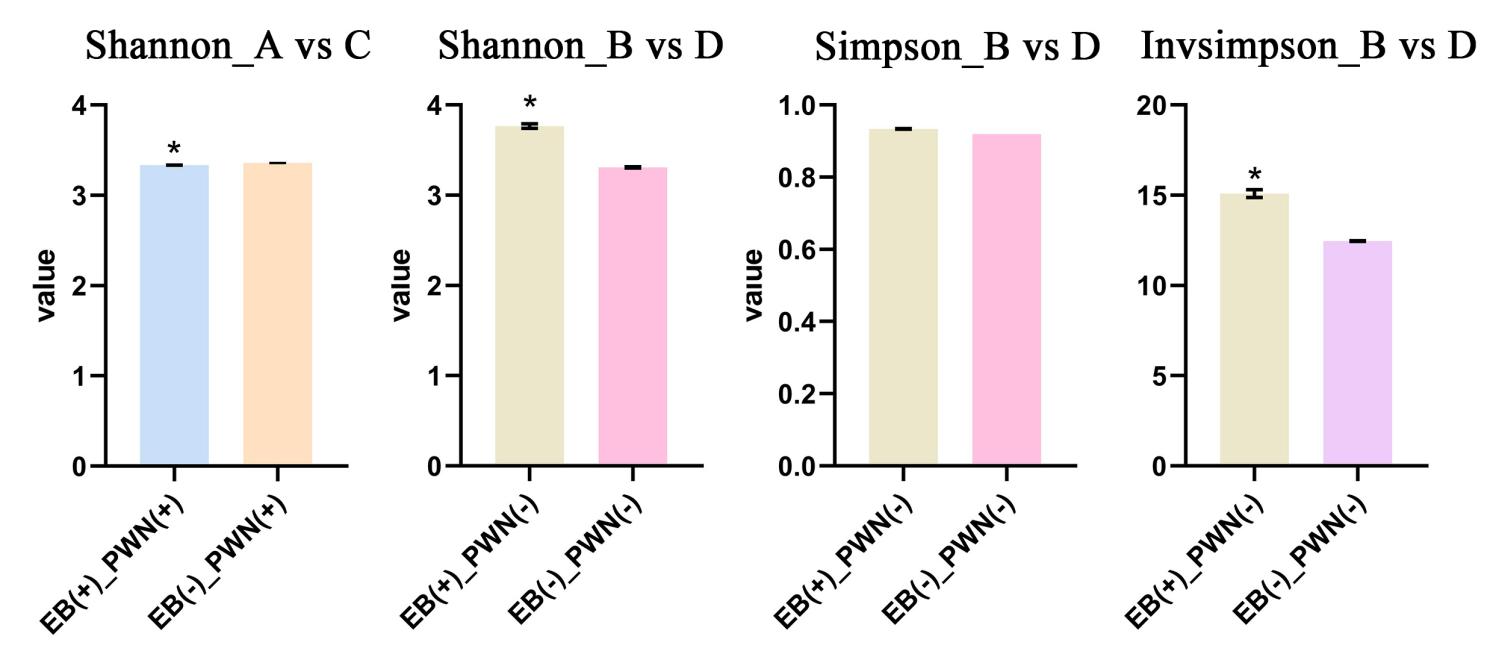


**Supplementary Figure S3. Effects of injection of EB (emamectin benzoate) on microbiote diversity of *Pinus massoniana.***

The impact of EB on symbiotic microbiota of *P. massoniana* seedling were presented by diversity of differential microbes. Sample A, B, C, D represents the seedlings carries both PWN and EB, EB only, PWN and control chemical, and control chemical only, respectively. EB (+) and EB (-) represent injected by EB or control chemical. PWN (+) and PWN (-) represent those host plant carries PWN or not. * represent significant differences between samples, *P* < 0.05, based on one-way ANOVA, with multiple comparison analysis using Tukey’s test.
