# Supplementary material for: The nematicide emamectin benzoate increases ROS accumulation in Pinus massoniana and poison Monochamus alternatus
Source: PLoS One. 2023 Dec 21;18(12):e0295945. doi: 10.1371/journal.pone.0295945 (PMC10735008; doi:10.1371/journal.pone.0295945)
Supplement: S6 Fig — (DOCX) [file pone.0295945.s008.docx]

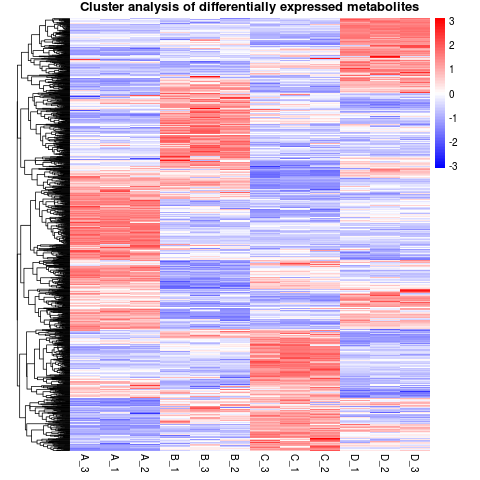


**Supplementary Figure S6. Effects of PWN injection on metabolites of host plants*.***

Hierarchical cluster analysis heat map of all differential metabolites regarding different treatment. Sample A, B, C, D represents the seedlings carries both PWN and EB, EB only, PWN and control chemical, and control chemical only, respectively.
