# Supplementary material for: The nematicide emamectin benzoate increases ROS accumulation in Pinus massoniana and poison Monochamus alternatus
Source: PLoS One. 2023 Dec 21;18(12):e0295945. doi: 10.1371/journal.pone.0295945 (PMC10735008; doi:10.1371/journal.pone.0295945)
Supplement: S7 Fig — (DOCX) [file pone.0295945.s009.docx]

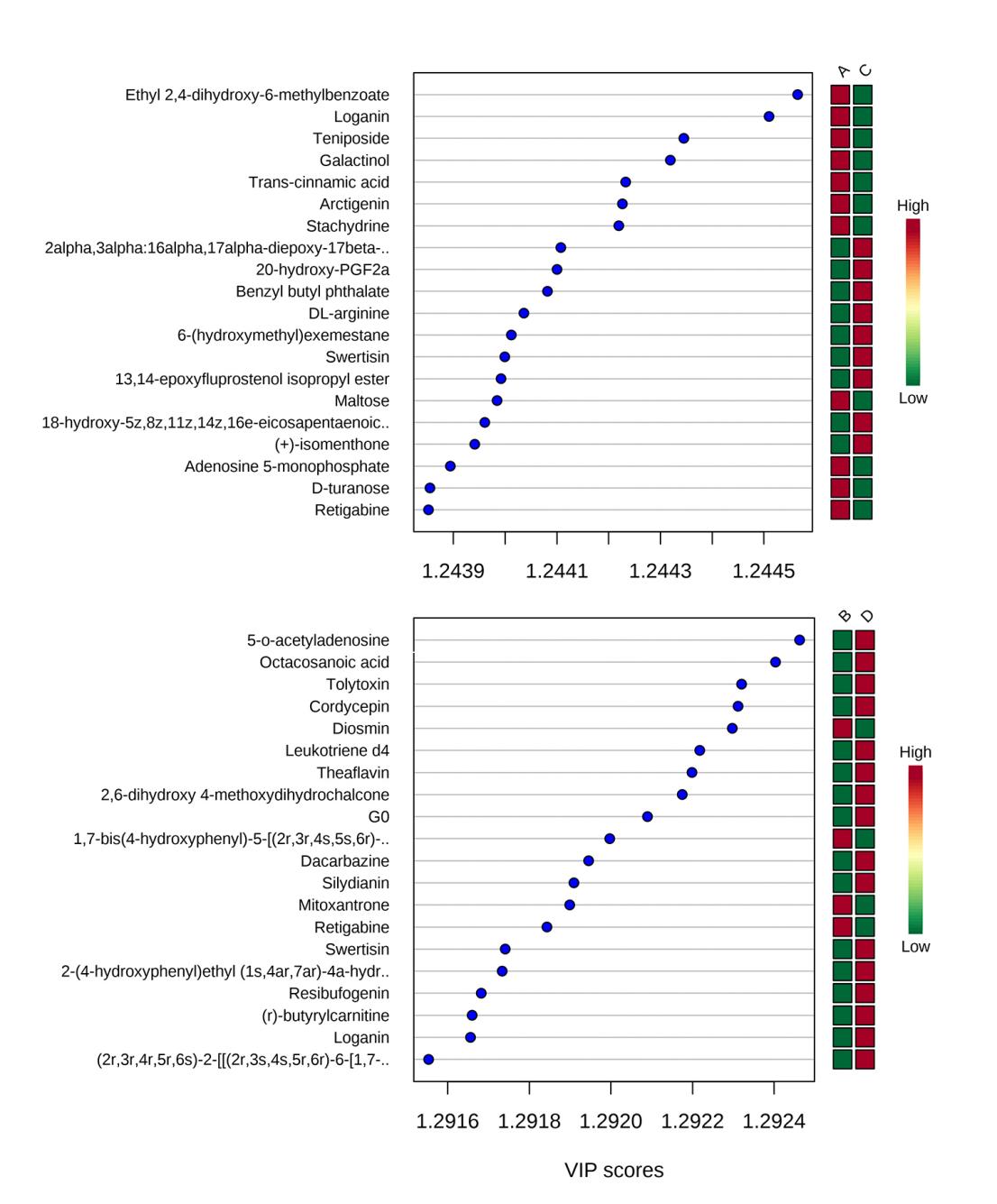


**Supplementary Figure S7. Effect of PWN injection on VIP of host plant*.***

VIP scatter diagram of different metabolites (showing the top 20 differential metabolites based on VIP value). The abscissa is the VIP value calculated by the OPLS-DA model. The color represents the expression pattern of differential metabolites. Sample A, B, C, D represents the seedlings carries both PWN and EB, EB only, PWN and control chemical, and control chemical only, respectively.
