# Supplementary material for: The nematicide emamectin benzoate increases ROS accumulation in Pinus massoniana and poison Monochamus alternatus
Source: PLoS One. 2023 Dec 21;18(12):e0295945. doi: 10.1371/journal.pone.0295945 (PMC10735008; doi:10.1371/journal.pone.0295945)
Supplement: S8 Fig — (DOCX) [file pone.0295945.s010.docx]

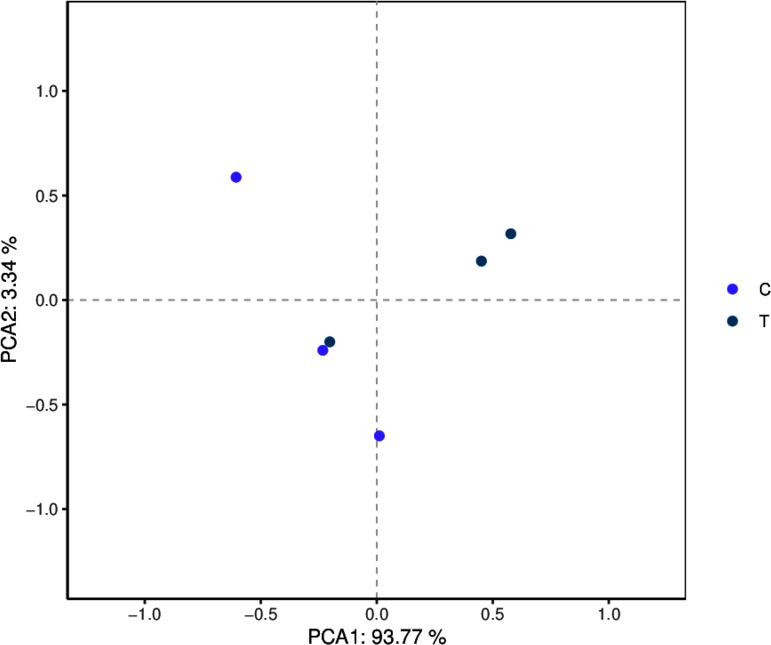


**Supplementary Figure S8. Principal component analysis of JPS feeding on intestinal microbes with or without EB injection.**

Principal component analysis of samples with different treatments. Each point in the diagram represents a sample, and different colors represent different groups. “T” and “C” represents JPS feed with seedlings contain EB and control chemical, respectively.
