# Supplementary material for: The nematicide emamectin benzoate increases ROS accumulation in Pinus massoniana and poison Monochamus alternatus
Source: PLoS One. 2023 Dec 21;18(12):e0295945. doi: 10.1371/journal.pone.0295945 (PMC10735008; doi:10.1371/journal.pone.0295945)
Supplement: S9 Fig — (DOCX) [file pone.0295945.s011.docx]

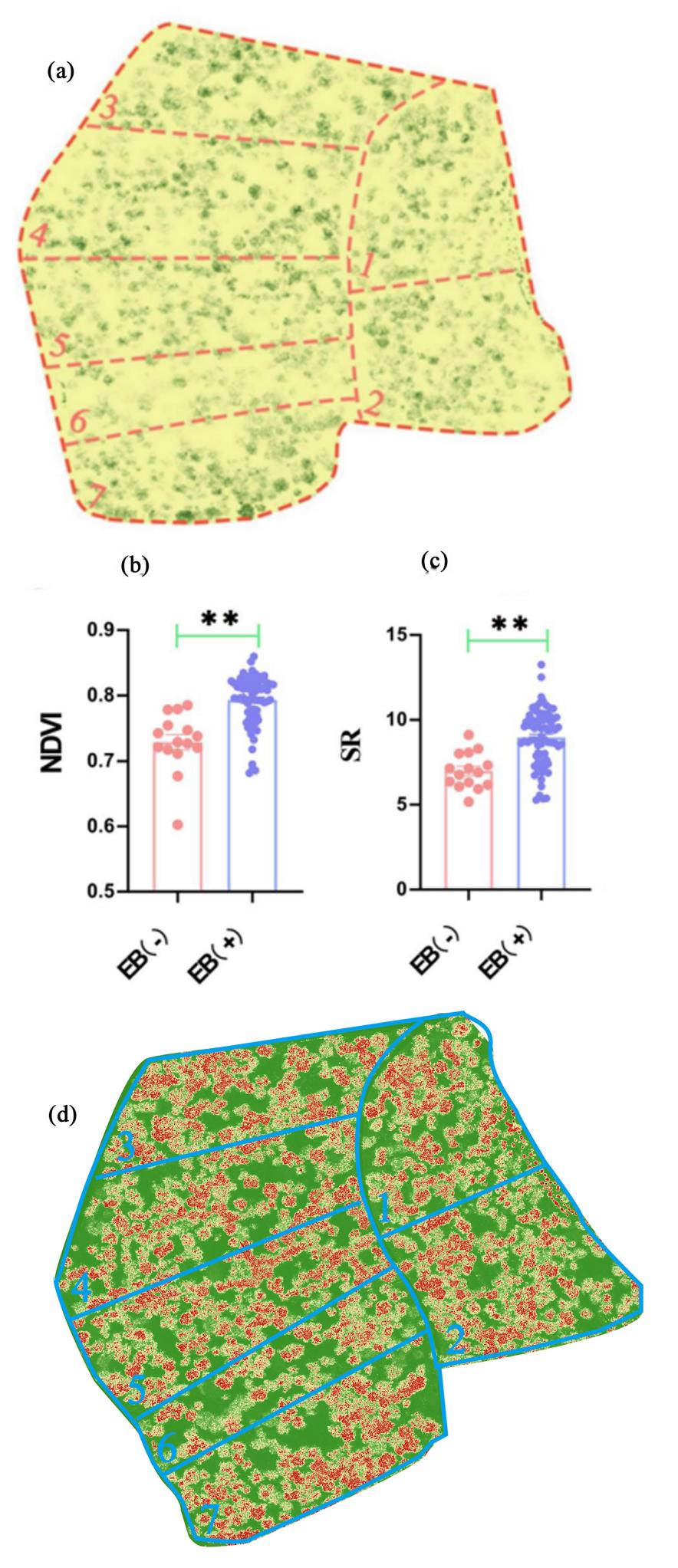


**Supplementary Figure S9. Effect of EB on chlorophyll content and water content of *Pinus massoniana*.**

UAV images of plots homogenized by vegetation index (a & d); normalized vegetation index (b) of plots injected with or without EB, and simple ratio index (c).* * represents significant difference between samples, P < 0.01, based on one-way ANOVA, with multiple comparison analysis using Tukey’s test.
